# Humans and Mice Display Opposing Patterns of “Browning” Gene Expression in Visceral and Subcutaneous White Adipose Tissue Depots

**DOI:** 10.3389/fcvm.2017.00027

**Published:** 2017-05-05

**Authors:** Maria A. Zuriaga, Jose J. Fuster, Noyan Gokce, Kenneth Walsh

**Affiliations:** ^1^Molecular Cardiology, Whitaker Cardiovascular Institute, Boston University School of Medicine, Boston, MA, USA; ^2^Cardiovascular Medicine, Whitaker Cardiovascular Institute, Boston University School of Medicine, Boston, MA, USA

**Keywords:** browning, adipose tissue, gene expression, mouse, human

## Abstract

Visceral adiposity is much more strongly associated with cardiometabolic disease in humans than subcutaneous adiposity. Browning, the appearance of brown-like adipocytes in the white adipose tissue (WAT), has been shown to protect mice against metabolic dysfunction, suggesting the possibility of new therapeutic approaches to treat obesity and type 2 diabetes. In mice, subcutaneous WAT depots express higher levels of browning genes when compared with visceral WAT, further suggesting that differences in WAT browning could contribute to the differences in the pathogenicity of the two depots. However, the expression of browning genes in different WAT depots of human has not been characterized. Here, it is shown that the expression of browning genes is higher in visceral than in subcutaneous WAT in humans, a pattern that is opposite to what is observed in mice. These results suggest that caution should be applied in extrapolating the results of murine browning gene expression studies to human pathophysiology.

Obesity is a major risk factor for cardiometabolic disease, and clinical studies have shown that different human adipose tissue depots contribute differentially to cardiometabolic risk. The accumulation of intra-abdominal visceral fat is a major contributor to systemic metabolic dysfunction that is strongly associated with cardiovascular risk in humans (1–3). In marked contrast, subcutaneous adipose tissue is benign or even protective with regard to cardiometabolic risk in some studies (4–6). An increasing body of evidence suggests that visceral and subcutaneous white adipose tissue (WAT) depots exhibit different intrinsic properties, which make visceral WAT a more pathogenic depot compared to subcutaneous WAT (7, 8). Surprisingly, the phenomenon of WAT “browning,” an actively investigated topic in the field of metabolism, remains ill defined in the context of human visceral and subcutaneous adiposity.

In contrast to WAT, brown adipose tissue (BAT) is a highly vascularized and has a high content of mitochondria (9). These mitochondria express high levels of uncoupling protein 1 (UCP1), allowing them to produce heat. In addition to this thermogenic function, BAT contributes significantly to systemic metabolism in rodent models because of its high energetic expenditure ratio (10–15). Recently, it has been appreciated that adult humans possess varying degrees of active BAT (16–19). Because BAT function will decrease with obesity and aging (17, 20), declining BAT function may link metabolic dysfunction and weight gain under these conditions.

“Browning” is the process by which some adipocytes within WAT depots acquire properties of brown adipocytes, i.e., an increase in mitochondrial content and oxidative capacity (21, 22). The browning of WAT occurs in response to various stimuli, including exposure to cold temperatures (23), β_3_-selective adrenergic agonists (24, 25), or exercise (26, 27). These adipocytes, termed “beige” or “brite” (brown in white), show an intermediate phenotype between white and brown adipocytes, exhibiting multilocular morphology, high levels of UCP1 expression, and some degree of thermogenic capacity. In addition, these cells have a distinct molecular signature, expressing markers, such as Tbx1, that are not present in white or brown adipocytes (28). Mouse studies have shown a correlation between WAT browning and decreased body weight gain and improved metabolic outcome when mice were challenged with obesogenic diets (21, 27, 29, 30). However, these systemic metabolic improvements could also be attributed to diminished BAT “whitening” and an increase in the BAT function under these conditions (9). Notably, studies have suggested the existence of major differences in the browning capacity of different WAT depots in mice (21, 30). In humans, browning has been observed in subcutaneous WAT of burned patients (31) and cancer cachexia (32); two conditions that are characterized by hypermetabolism, large increases in resting energy expenditure and whole-body catabolism and associated with increased morbidity and mortality. Thus, more information about the browning potential of human WAT depots and its metabolic implications is necessary to be able to extrapolate the effects observed in mice.

The brown adipose determination factor Prdm16 has been reported to play a central role in the browning capacity of WAT. Prdm16 is differentially expressed in various mouse WAT depots, and this differential expression has been suggested to mediate differences in their browning capacity. Specifically, the higher expression of Prdm16 and other BAT-selective genes has been shown to correlate with the higher browning susceptibility of subcutaneous (inguinal) WAT compared to the visceral (epididymal) WAT in lean wild-type mice (21). Consistently, subcutaneous WAT in adipose tissue-specific Prdm16-deficient mice adopts visceral WAT-like qualities and exhibits decreased browning capacity, and this correlates with greater weight gain and insulin resistance (30). These mouse studies suggest that differences in browning susceptibility could contribute to the intrinsic differences between WAT depots that determine their differential impacts on cardiometabolic risk (33, 34). However, previous studies have almost exclusively focused on the murine system, and the expression of Prdm16 and other browning-related genes in human WAT depots has not been explored.

In this study, we compared the expression of browning genes in subcutaneous and visceral WAT collected from obese human individuals and chronically obese mice to gain insight into the susceptibility of different WAT depots to browning in a context of chronic obesity. Consistent with previously published data in lean mice (21), subcutaneous (inguinal) WAT in obese mice displayed higher transcript levels of the browning markers UCP1, Cidea, and Pdrm16, and the beige adipocyte markers Tbx1 and P2rx5, compared with their expression levels in visceral (epididymal) WAT (Figure 1A). Correspondingly, the mitochondrial genes Cox8b, Ppargc1a, Atp5a, and Ndufa1 are more highly expressed in inguinal compared to epididymal WAT. These mouse data are consistent with the prevailing notion that subcutaneous WAT is more susceptible to browning than visceral WAT.

**Figure 1:**
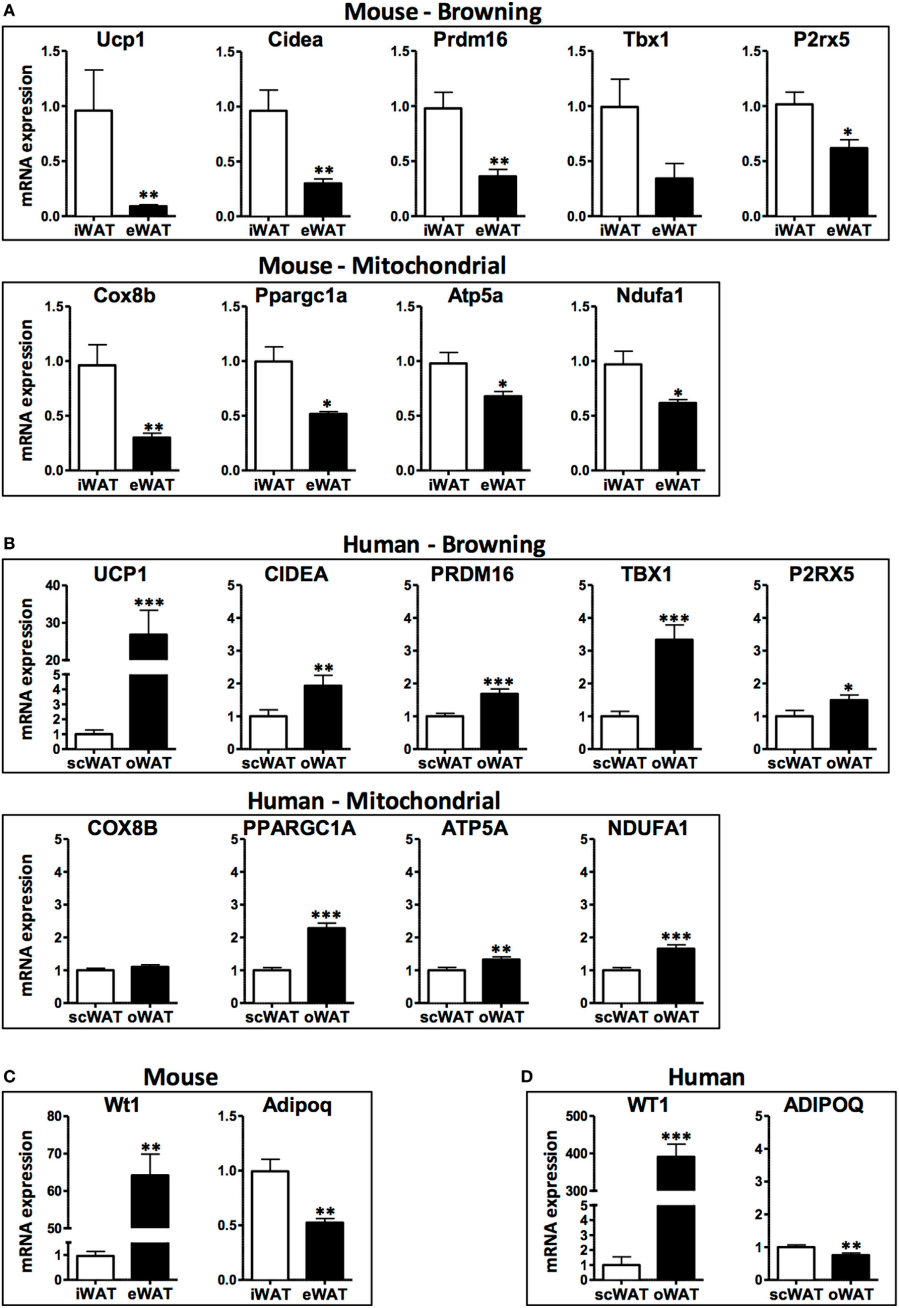
**mRNA levels in subcutaneous and visceral white adipose tissue (WAT) depots of obese mice and humans**. **(A)** Browning and mitochondrial genes in iWAT (inguinal, subcutaneous) and eWAT (epididymal, visceral) depots of mice, average *C*_t_ values: Ucp1: 27.1; Cidea: 27.9; Prdm16: 28.6; Tbx1: 35.0; P2rx5: 28.4; Cox8b: 22.5; Ppargc1a: 27.2; Atp5a: 19.7; Ndufa1: 21.3 and **(B)** scWAT (subcutaneous, abdominal region) and oWAT (omental, visceral) depots of humans, average *C*_t_ values: UCP1: 28.8; CIDEA: 18.3; PRDM16: 26.5; TBX1: 23.1; P2RX5: 23.6; COX8B: 20.1; PPARGC1a: 21.8; ATP5a: 15.9; NDUFA1: 18.3. **(C)** Visceral marker Wt1 and adipokine adiponectin in iWAT and eWAT depots of mice, average *C*_t_ values: Wt1: 30.5; Adipoq: 18.2 and **(D)** scWAT and oWAT depots of humans, average *C*_t_ values: WT1: 24.0; ADIPOQ: 13.6. Samples were collected from: C57BL6/J mice (*n* = 9) fed a high fat and high sucrose diet for 30 weeks and housed at 21°C (average body weight: 53.5 g, 40.6% weight increase compared to lean controls); and obese humans (*n* = 21–32) at the time of bariatric surgery, clinical characteristics detailed in Table 1. RNA was isolated using RNeasy Lipid Tissue Mini Kit (Qiagen), and synthesis of cDNA was performed with high capacity cDNA Reverse Transcription Kit (Applied Biosystems). Quantitative real-time PCR reactions were performed in a ViiA7 System (Applied Biosystems) using TaqMan gene expression assays (Applied Biosystems) for the human samples and SYBR Green-based assays for the murine samples (primer sequences obtained from http://mouseprimerdepot.nci.nih.gov).

**Table 1:** **Clinical characteristics of human population studied (*n* = 32)**.

Clinical parameters	
Age (years)	42 ± 2
Female sex (%)	75
BMI (kg/m^2^)	43 ± 1
Weight (kg)	122 ± 5
Waist circumference (cm)	132 ± 3
Waist to hip ratio	0.97 ± 0.02
Diabetes (%)	37
Fasting glucose (mg/dl)	130 ± 13
Plasma insulin (mIU/ml)	18 ± 3
HOMA	5.5 ± 0.9
Triglycerides (mg/dl)	118 ± 13
Hypercholesterolemia (%)	22
Total cholesterol (mg/dl)	170 ± 7
LDL cholesterol (mg/dl)	105 ± 5
HDL cholesterol (mg/dl)	41 ± 1
hsCRP (mg/dl)	9.4 ± 1.3
Hypertension (%)	56
Systolic blood pressure (mm Hg)	126 ± 2
Diastolic blood pressure (mm Hg)	73 ± 1
Coronary heart disease (%)	6

*Data expressed as mean ± SEM*.

In contrast, the analysis of human WAT specimens suggests a markedly different scenario (Figure 1B). Compared with subcutaneous WAT, visceral (omental) WAT exhibits higher transcript levels of UCP1 and all of the browning genes examined in this study (CIDEA, PRDM16, TBX1, and P2RX5). Consistently, human visceral WAT displayed higher levels of the mitochondrial gene transcripts COX8B, PPARGC1A, ATP5A, and NDUFA1. Overall, these data suggest that human visceral fat is more likely to exhibit a higher browning capacity than subcutaneous fat.

When interpreting the opposite browning gene expression patterns in WAT depots from human and mouse, it must be considered from the data presented herein, as most studies comparing data on adipose tissue from mice and humans, that the “visceral” designation actually involves a comparison of different intra-abdominal WAT depots. In this regard, the human omental fat depot is essentially non-existent in mice. Furthermore, the mouse epididymal or perigonadal fat depot does not drain into the portal circulation and therefore is technically not a true visceral depot. Despite these anatomical differences, mouse subcutaneous-epididymal characteristics frequently track with human subcutaneous-omental characteristics. For example, the expression of the anti-inflammatory adipokine adiponectin (ADIPOQ/Adipoq) is decreased in visceral depots relative to subcutaneous fat in both human and mice, and the transcript levels of the visceral WAT marker Wilms tumor 1 (WT1/Wt1) (30) are increased in both mouse epididymal fat and human omental fat (Figures 1C,D). Furthermore, the expression of various pro-inflammatory molecules is similarly elevated in both human and mouse visceral WAT depots compared to subcutaneous WAT (35–38).

The issue of thermoneutrality should be considered when analyzing the browning process in mice and humans. Humans have replaced the endogenous thermoregulatory mechanisms with the donning of clothing and macro-environmental manipulations (39). However, mice are commonly housed at temperatures below their thermoneutral range, leading to mild cold stress that can affect many biological processes (40, 41). For example, cold acclimatization is associated with large increases in UCP1 gene expression in beige adipose tissue in mice housed at 21°C (common conditions), but this increase is not observed in mice housed at 30°C (thermoneutrality) (42). Accordingly, mice housed at 21°C (including the mice used in this study) exhibit a perpetual activation of brown and beige adipose tissues.

Conflicting findings of mouse and human studies in the setting of browning have been reported by other groups. WAT browning has not been observed in response to exercise in humans (43), in contrast to mice (26, 27). In addition, a study of combined diet and exercise weigh loss intervention in women did not result in a higher beige adipocyte induction, but in a decrease in UCP1 expression and a remodeling of scWAT toward a more-white phenotype instead of a more-brown phenotype (44). These studies suggest that human adipose tissue does not respond to browning stimuli identified in mouse models.

Overall, our findings suggest that mouse epididymal fat mimics some of the pathogenic properties of human visceral fat, but it is not a valid surrogate for human visceral fat in the context of browning. Since most of the studies comparing subcutaneous and visceral depots in mice and humans use these WAT depots, we urge caution when extrapolating results from mice to humans. In the case of WAT browning, differences in gene expression patterns between fat depots cannot be extrapolated from mouse to humans. Our data also suggest that it is unlikely that the increased cardiometabolic risk associated with visceral adiposity in humans is due to the reduced browning capacity of visceral adipose tissue.

## Ethics Statement

The human study was approved by Boston Medical Center Institutional Review Board, and all subjects gave written informed consent. The animal study was approved by the Institutional Animal Care and Use Committee at Boston University Medical Campus.

## Author Contributions

MZ acquired and analyzed the data. MZ and JF drafted the paper. NG and KW provided supervision and funding and critical revisions to the manuscript.

## Conflict of Interest Statement

The authors declare that the research was conducted in the absence of any commercial or financial relationships that could be construed as a potential conflict of interest.
